# Insights into the Epidemiology, Phylodynamics, and Evolutionary Changes of Lineage GI-7 Infectious Bronchitis Virus

**DOI:** 10.1155/2023/9520616

**Published:** 2023-05-16

**Authors:** Libin Chen, Weiwei Jiang, Wanyan Wu, Siyuan Zhang, Juncheng Cai, Ting Lv, Bin Xiang, Qiuyan Lin, Ming Liao, Chan Ding, Tao Ren

**Affiliations:** ^1^College of Veterinary Medicine, South China Agricultural University, Guangzhou 510642, China; ^2^Key Laboratory of Animal Vaccine Development, Ministry of Agriculture, Guangzhou 510642, China; ^3^National and Regional Joint Engineering Laboratory for Medicament of Zoonosis Prevention and Control, Guangzhou 510642, China; ^4^Key Laboratory of Zoonosis Prevention and Control of Guangdong Province, Guangzhou 510642, China; ^5^Guangzhou South China Biological Medicine Co., Ltd., Guangzhou 510642, China; ^6^College of Veterinary Medicine, Yunnan Agricultural University, Kunming 650201, China; ^7^Key Laboratory for Prevention and Control of Avian Influenza and Other Major Poultry Diseases, Ministry of Agriculture and Rural Affairs, Guangzhou 510642, China; ^8^Shanghai Veterinary Research Institute (SHVRI), Chinese Academy of Agricultural Sciences (CAAS), Shanghai 200241, China

## Abstract

Infectious bronchitis virus (IBV) is distributed worldwide and causes significant losses in the poultry industry. In recent decades, lineages GI-19 and GI-7 have become the most prevalent IBV strains in China. However, the molecular evolution and phylodynamics of the lineage GI-7 IBV strains remain largely unknown. In this study, we identified 19 IBV strains from clinical samples from January 2021 to June 2022 in China, including 12 strains of GI-19, 3 strains of GI-7, and 1 strain each of GI-1, GI-9, GI-13, and GI-28. These results indicated that lineages GI-19 and GI-7 IBVs are still the most prevalent IBVs in China. Here, we investigated the evolution and transmission dynamics of lineage GI-7 IBVs. Our results revealed that the Taiwan province might be the origin of lineage GI-7 IBVs and that South China plays an important role in the spread of IBV. Furthermore, we found low codon usage bias of the S1 gene in lineage GI-7 IBVs. This allowed IBV to replicate in the host during evolution as a result of reduced competition, mainly driven by natural selection and mutational pressure, where the role of natural selection is more prominent. Collectively, our results reveal the genetic diversity and evolutionary dynamics of lineage GI-7 IBVs, which could assist in the prevention and control of viral infection.

## 1. Introduction

Since the outbreak of coronavirus disease (COVID-19) caused by severe acute respiratory syndrome coronavirus 2 (SARS-CoV-2) in late 2019, the world economy and human health have suffered significantly damage, drawing further attention to the complex and huge coronavirus family [1, 2]. The most severely affected coronavirus in the poultry industry is the infectious bronchitis virus (IBV), which can cause infectious bronchitis (IB) in chickens of all ages and has caused huge economic losses worldwide [3]. The prevention and control of IB are still current challenges.

IBV is a positive RNA single-stranded virus; however, unlike SARS-CoV-2, which belongs to the *β*-coronavirus family, IBV belongs to the *γ*-coronavirus family [4]. The IBV genome encodes four structural proteins: nucleocapsid (*N*) protein, envelope (*E*) protein, membrane (*M*) glycoprotein, and spike (*S*) glycoprotein, among which the *S* protein must be cleaved into the *S*1 and *S*2 subunits for viral replication [5]. Unlike the conserved *S*2 subunit, the *S*1 subunit contains IBV viral neutralizing epitopes and three different hypervariable regions (HVRs; AA 38–67, 91–141, and 274–387) that play a critical role in IBV virulence and tissue tropism [6, 7]. Therefore, analysis of the nucleotide sequence encoded by the *S*1 gene has been conventionally used to determine the genetic type of IBV. Among the numerous classifications based on the *S*1 gene, the most commonly used currently divides IBV into at least 35 lineages, including 7 genotypes (GI–GVII), with many additional interlineage recombinants [8, 9].

IBV has been highly prevalent in China for a long time, with a wide geographical infection range; this has been harmful to the development of the poultry industry. Since its first isolation in 1996, lineage GI-19 (QX-type) has become the most prevalent IBV strain in China in the recent decade [10, 11]. Moreover, lineages GI-7 (TW-type) and GI-13 (4/91-type) have also been reported as major IBV strains in China, with the lineages GI-1 (Mass-type), GI-9 (Ark-type), and GI-28 (LDT3-type) reported sporadically [12, 13]. The isolation rate of lineage GVI-1 IBV strains has also increased in recent years [14–16], as part of critical continuous epidemiological surveillance of the complex prevalence of IBV in China.

Bayesian phylogeographic inference is widely accepted in the study of the evolutionary history and spatiotemporal dynamics of RNA viruses using genomic and geospatial data [17-20]. This method has also been used to investigate the evolution and transmission of the GI-19 IBV lineage [21], which was first isolated in the Taiwan province of China in 1965, and is known as the TW type and divided into TW-I and TW-II sublineages [22]. As one of the main circulating IBVs in China, the threats and number of lineage GI-7 IBV isolates have increased gradually in recent years [11, 23]. However, the molecular evolution and phylodynamics of the lineage GI-7 IBV strains remain largely unknown.

Synonymous codons are not randomly chosen within and between genomes, a phenomenon called codon usage bias [24, 25] that allows viruses to efficiently survive and adapt to their hosts [26]. Codon usage patterns are influenced by natural or translational selection and mutation pressure. Many RNA viruses have a low codon usage bias [27–30] that allows for efficient replication in the host cell by lowering competition with host genes. Therefore, analysis of IBV codon usage patterns could provide insights into the evolution of IBV.

In this study, we conducted an epidemiological surveillance of IBV in China from January 2021 to June 2022 and identified the S1 gene of 19 IBV strains isolated from clinical samples. We also investigated the prevalence, genotype, and recombination of these viruses. In addition, we explored the molecular evolution, phylogeography, and codon usage patterns of all GI-7 IBVs isolated.

## 2. Materials and Methods

### 2.1. Virus Isolation and Identification

IBV strains were isolated from tissue samples of chickens with clinical respiratory symptoms raised on poultry farms in China during an active surveillance program between January 2021 and June 2022. All sick chickens were immunized against mass strains such as H120 and H52. The samples were treated as previously described [14]. The presence of IBVs was verified, and the *S*1 gene of the isolates was sequenced via reverse transcription polymerase chain reaction (RT-PCR), as described previously [14].

### 2.2. Recombination Analysis

Putative recombination events and parental strains were identified using the Recombination Detection Program version 4.0 (RDP 4.0; Simmonics, University of Warwick, Coventry, UK). Multiple methods and default program settings were used to analyze the data, including RDP, Bootscan, GeneConv, Maxchi, Chimaera, SiSscan, LARD, 3Seq, and PhylPro [31]. The most likely recombinant fragments (*p* ≤ 10^−12^) were determined along with the possible parental virus and the beginning and end points. The potential recombination events and breakpoints were determined by similarity plot (SimPlots) analysis in SimPlot version 3.5.1, using a window of 200 bp and a step size of 20 bp.

### 2.3. Phylogenetic Analysis

In addition to the IBVs isolated in this study, 107 *S*1 gene sequences of 35 lineage IBV strains were obtained from GenBank (NCBI, Bethesda, MD, USA). All sequences were aligned using MAFFT v7.221.3 [32]. A maximum likelihood (ML) tree was constructed based on the *S*1 gene sequences and GTR + *F* + *R*5 model, selected with ModelFinder [33], using IQ-TREE software [34] with 1000 bootstrap replicates. The ML tree was visualized using Figtree version 1.4.2.

To determine the temporal structure of the lineage GI-7 IBV strains, in addition to the IBVs isolated in this study, all 199 *S*1 gene sequences (*n* ≥ 1620 nucleotides, nt) of lineage GI-7 IBVs submitted before August 2022 were collected from GenBank (Table S1) and divided into seven regions according to geographical location. A regression of root-to-tip genetic distance was performed for the data set using TempEst software [35] based on the unrooted ML tree of lineage GI-7 IBVs generated by IQ-TREE and implemented by the TIM + *F* + *R*5 model selected in ModelFinder. The Bayesian Markov chain Monte Carlo (MCMC) method was used to infer the evolutionary rate and timescale of lineage GI-7 IBVs in BEAST version 1.10.4 [36], with a strict clock model and constant-size coalescence. The MCMC was run in parallel for four chains with the GTR + *F* + *G*4 substitution model selected using ModelFinder, each with 100 million steps and a burn-in of 10%. The parallel result files were integrated using LogCombiner (part of BEAST version 1.10.4). Convergence of all parameters (i.e., effective sample sizes >200) was confirmed visually using Tracer version 1.7 [37]. The maximum clade credibility (MCC) tree was inferred using TreeAnnotator (part of BEAST version 1.10.4) and visualized using FigTree version 1.4.2. A similar statistical method was used to estimate the most recent common ancestor (TMRCA) for sublineages TW-I and TW-II of the GI-7 IBV strains. To investigate the demographic history of all lineage GI-7 IBV strains, a Bayesian skyline plot (BSP) was used to infer changes in lineage GI-7 IBV in an effective population size, using BEAST version 1.10.4 [18]. The results were plotted using Tracer version 1.7 [37].

### 2.4. Bayesian Phylogeographic Analysis

To understand the spatial diffusion patterns of the GI-7 IBVs, an asymmetric continuous-time Markov chain phylogeographic model with Bayesian stochastic search variable selection (BSSVS) was implemented using BEAST version 1.10.4. The ancestral geographical regions, diffusion rates, and migration patterns of these viruses were then analyzed. GI-7 IBV strains from different regions of China were selected and coded as discrete states using a strict clock model and Bayesian skyline coalescence. We also applied a BSSVS procedure to identify the best-supported individual transitions between the discrete states. The Bayes factor (BF) test was adopted to identify significant nonzero transition rates in SPREAD3 version 0.9.7 [38]. To confirm the reliability of the analysis, the MCMC was run five times, independently, each with 100 million steps and a burn-in of 10%. Significant transitions were determined based on the combination of both BF ≥ 3 and a mean indicator of 0.5, where 3 ≤ BF < 10 indicated statistically significant support, 10 ≤ BF < 100 indicated strong support, 100 ≤ BF < 1000 indicated very strong support, and BF ≥ 1000 indicated decisive support [17].

### 2.5. Codon Usage Analysis

To understand the codon usage patterns in the evolution of lineage GI-7 IBVs, we investigated the codon usage bias patterns of IBVs from two sublineages, TW-I and TW-II. Furthermore, we determined the frequencies of all nucleotides (*A*%, *U*%, *G*%, *C*%, AU%, and GC%) as well as the *A*, *C*, *G*, and *U* frequencies of codons at different sites (GC1%, GC2%, GC3%, GC12%, *A*3%, *U*3%, G3%, *C*3%, and AU3%). The nucleotide frequencies of synonymous codons at the third position and the effective number of codons (ENC) were calculated using Codon W v1.4.2 (https://codonw.sourceforge.net/).

To identify the most commonly used synonymous codons, the relative synonymous codon usage (RSCU) values for 59 codons were calculated using MAFFT v7.221.3. RSCU values less than, greater than, and equal to 1.0 represented negative codon usage bias, positive codon usage bias, and no bias, respectively [39, 40]. Codons with RSCU <0.6 and >1.6 were considered under- and over-represented, respectively [41].

To investigate the impact of mutation and selection pressure on codon usage, parity rule 2 (PR2) plot analysis was performed with GC deviation [*G*3/(*G*3+*C*3)] as the abscissa and AU deviation [*A*3/(*A*3+*U*3)] as the ordinate. The genome is evenly distributed in the center of the graph when *A* = *U*, *G* = *C*, indicating that mutation pressure and selectivity (substitution rate) have the same effect on codon usage [42].

To investigate the effects of natural selection and mutation pressure on codon usage bias, a neutrality analysis was performed using GC3s as the horizontal coordinate and GC12 as the vertical coordinate. The GC3s and GC12 contents of *S*1 genes of lineage GI-7 IBVs in the dataset were plotted, and the regression line was calculated. Regression lines with a slope close to 1 indicate that the genome is distributed almost diagonally and that codon usage bias is only affected by mutation pressure, while a decrease in the slope indicates an increase in the effect of natural selection [29, 43].

## 3. Results

### 3.1. Virus Identification and Phylogenetic Analysis

In this study, 591 samples were collected from poultry farms in China between January 2021 and June 2022. A total of 19 (19/591, 3.21%) IBV strains were isolated and identified, including 9 isolates from Guangdong province (9/19, 47.37%), 4 from Guangxi province (4/19, 21.05%), 3 from Yunnan province (3/19, 15.79%), and 1 each from Hebei province (1/19, 5.26%), Shandong province (1/19, 5.26%), and Jiangxi province (1/19, 5.26%) (Table S2).

**Figure 1:**
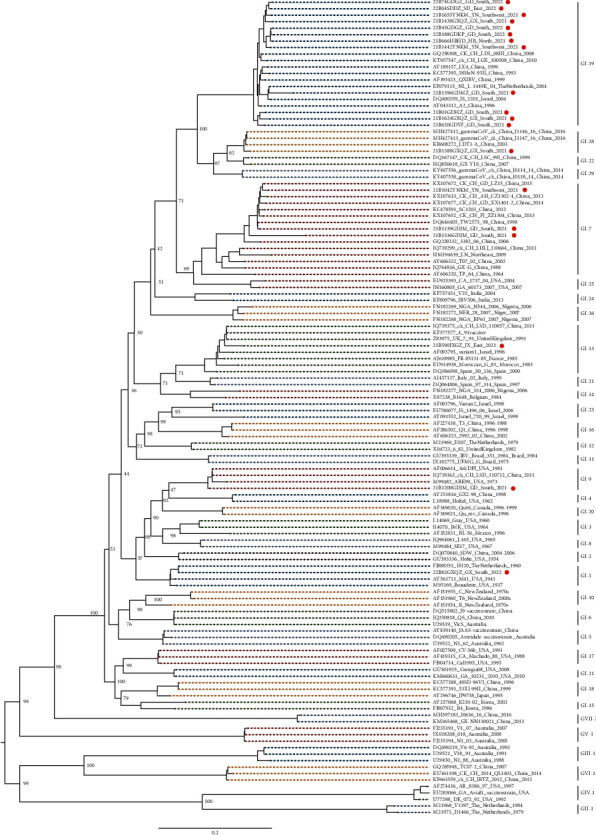
Maximum likelihood (ML) tree based on the *S*1 gene from 35 lineages of infectious bronchitis virus (IBV). The tree was constructed using IQ-TREE software with the GTR + *F* + *R*5 model. Red circles indicate the stains isolated in this study.

Based on the *S*1 gene sequences, an ML tree was constructed, and the 19 isolated IBV strains segregated into 6 lineages: GI-19 (12/19, 63.16%), GI-7 (3/19, 15.79%), GI-1 (1/19, 5.26%), GI-9 (1/19, 5.26%), GI-13 (1/19, 5.26%), and GI-28 (1/19, 5.26%) (Figure 1).

### 3.2. Recombination Analysis

**Figure 2:**
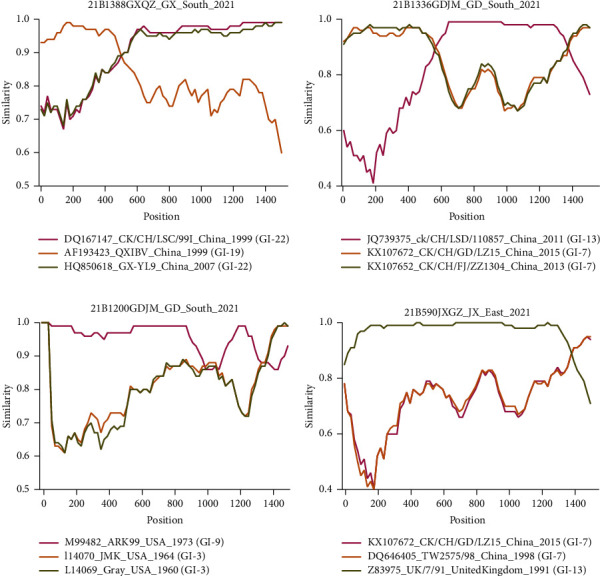
Recombination events in the *S*1 gene from the isolated infectious bronchitis virus (IBV) strains. Simplot analysis was performed to detect recombination within the *S*1 gene from (a) 21B1388GXQZ, (b) 21B1336GDJM, (c) 21B1200GDJM, and (d) 21B590JXGZ. The *y*-axis represents the ratio of identity within a 200-bp wide sliding window centered on the position plotted, with a 20 bp step size between plots.

Recombination analysis of the *S*1 gene sequences of all 19 IBV strains isolated in this study was performed using RDP 4.0. The four isolates, 21B1388GXQZ (Figure 2(a)), 21B1336GDJM (Figure 2(b)), 21B1200GDJM (Figure 2(c)), and 21B590JXGZ (Figure 2(d)), were found to have recombination events. The breakpoint positions and specific *p* values for each recombination event detection method are listed in Table S3. As shown in Figures 2(a)–2(d), recombination of multiple IBV lineages was observed, and lineage GI-7 and GI-13 IBVs were identified as parental strains in two recombination events. Together, these results indicated that prevalent lineage IBVs, such as GI-7 strains, have a higher probability of involvement in recombination events.

### 3.3. Population and Evolutionary Dynamics of Lineage GI-7 IBVs

**Figure 3:**
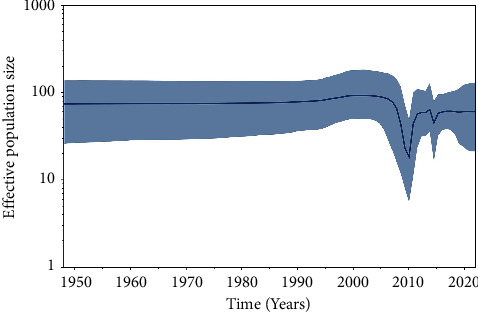
Bayesian skyline plot (BSP) of the *S*1 gene from lineage GI-7 infectious bronchitis virus (IBV) strains. The dark blue line indicates the mean value of genetic diversity, and the light blue shading shows the 95% confidence interval.

Owing to the increasing threat of lineage GI-7 IBVs, we collected all *S*1 gene sequences of this lineage submitted to GenBank before August 2022 for analysis. The Bayesian skyline coalescent was reconstructed to reveal the relative genetic diversity of GI-7 IBVs and illustrate the effective population size of these viruses. The population size of lineage GI-7 IBVs was relatively constant prior to 2007, with a large reduction in the population size observed after 2007, indicating a decrease in the relative genetic diversity of these viruses (Figure 3). The population size of lineage GI-7 IBVs expanded after 2010, fluctuated around 2015, and then exhibited a steady state.

### 3.4. Spatial Dynamics of Lineage GI-7 IBVs

**Figure 4:**
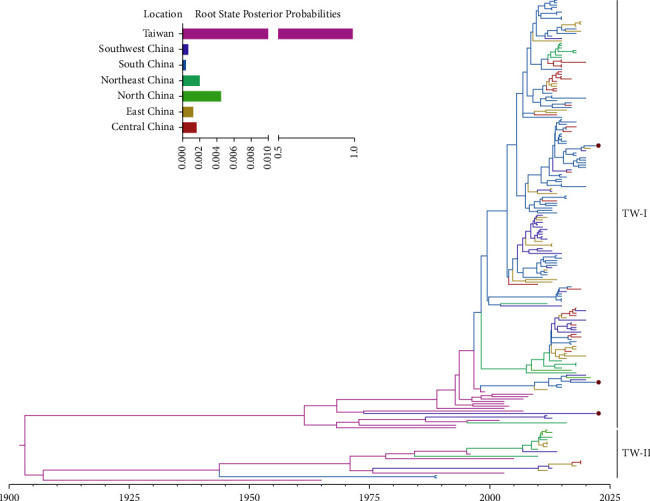
Maximum clade credibility (MCC) tree of the *S*1 gene from lineage GI-7 infectious bronchitis virus (IBV) strains. The trees were constructed using BEAST version 1.10.4 software. All lineage GI-7 IBV strains contained the 202 isolates available in GenBank as of August 2022, with the three isolates identified in our study indicated by red circles. The root state posterior probabilities for the regions are shown in the inset panel.

A root-to-tip regression analysis of lineage GI-7 IBVs showed that the correlation coefficient and *R*^2^ were 0.6629 and 0.4394, respectively, confirming the presence of a temporal structure. The time-scaled MCC tree of lineage GI-7 IBVs based on the *S*1 gene showed that all lineage GI-7 IBVs were divided into two sublineages, TW-I and TW-II and that all three strains isolated in this study belonged to sub-lineage TW-I (Figure 4). The majority of lineage GI-7 IBVs belonged to sublineage TW-I, with TMRCA occurring in 1947 (95% highest posterior density (HPD): 1935–1959). Furthermore, the first lineage GI-7 IBV isolate, TP/64, was identified as sublineage TW-II, with TMRCA occurring in 1886 (95% HPD: 1865–1906). In addition, the Bayesian analysis placed the root of the tree in the Taiwan province, with a posterior probability of 0.98 (Figure 4).

**Figure 5:**
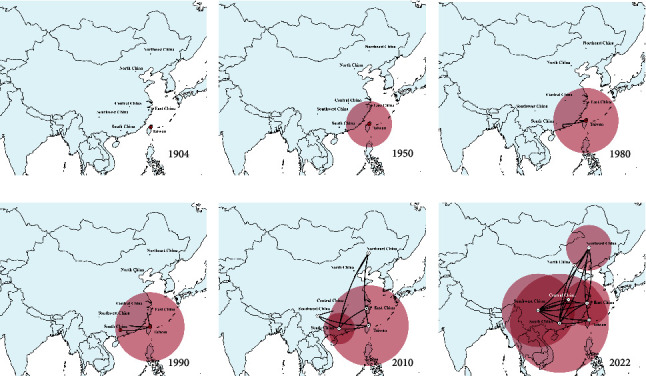
Spatiotemporal dynamics of lineage GI-7 infectious bronchitis virus (IBV) strains among the different localities tested. Snapshots of dispersal patterns in (a) 1904, (b) 1950, (c) 1980, (d) 1990, (e) 2010, and (f) 2022. The data were collected from GenBank that were submitted before June 2022 and from this study.

The dispersal history of lineage GI-7 IBVs was determined via global animations made in SpreaD3. Snapshots of dispersal patterns also showed that the Taiwan province was the origin of the lineage GI-7 IBVs in the early 1900s (Figure 5(a)). This lineage then propagated outwards to South and Southwest China after the 1950s and 1980s, respectively (Figures 5(b) and 5(c)), and was then transmitted to South China from Taiwan by the early 1990s (Figure 5(d)). The lineage GI-7 IBVs from South, Southwest, and East China spread outwards in the 2010s (Figure 5(e)) and have become prevalent throughout China in recent years (Figure 5(f)).

**Figure 6:**
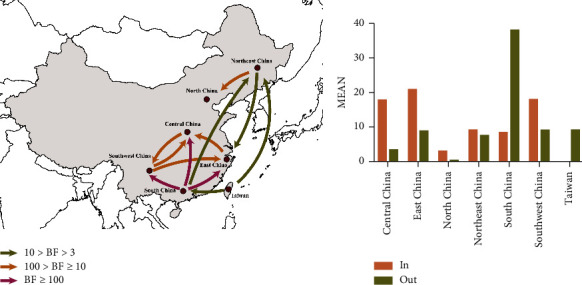
Spatial diffusion of lineage GI-7 infectious bronchitis virus (IBV) strains. (a) Spatial diffusion pathways of lineage GI-7 IBV strains. Only statistically supported migrations with a BF > 3 are shown. (b) Histogram of the total number of state transitions.

The circulation of lineage GI-7 IBVs was estimated by performing BSSVS analysis, which supported the presence of 12 migration links in the diffusion of lineage GI-7 IBVs (Figure 6(a); Table 1). The lowest mean migration rates were observed from Northeast China to North China, whereas the highest mean migration rates were observed from South China to Southwest China (Table 1). Three routes from South China were identified with decisive support, whereas one route from South China was identified with statistically significant support. In addition, the routes from Taiwan province to Northeast and South China were identified with statistically significant support, and several routes from East, Northeast, Southwest, and Central China were also identified. Furthermore, migration from South China was much greater than that from the other regions (Figure 6(b)). These results revealed that South China might have played a key role in causing the lineage GI-7 IBVs epidemic in China.

### 3.5. Codon Usage Patterns of *S*1 Genes in Lineage GI-7 IBVs

Codon usage analysis was performed to further explore the molecular evolution of lineage GI-7 IBVs. Nucleotide and synonymous codon composition analyses showed that *A* (27.715 ± 2.688%) and *U* (35.881 ± 2.723%) were used more frequently than *C* (16.903 ± 5.904%) and *G* (19.499 ± 2.631%) in *S*1 genes of lineage GI-7 IBVs, with the majority of codons ending in A/U (76.79%) (Table S1). Moreover, the nucleotide content of the synonymous codons at the third position was U3s > A3s > G3s > C3s. The RSCU value further confirmed that the frequency of *S*1 gene codons ending in *U*/*A* was higher than those ending in *C*/*G*. Among the 18 preferred synonymous codons of the *S*1 gene of lineage GI-7 IBVs, 16 ended with *A*/*U*, whereas only 2 ended with *C*/*G*. Analysis of codon over- and under-representation revealed that 8 out of the 18 preferred codons had RSCU values >1.6, including GUU (V), UUA (L), AUU (I), UCU (S), GCA (A), AGG/A (R), CCU (P), and GGU (G) (Table S4).

**Figure 7:**
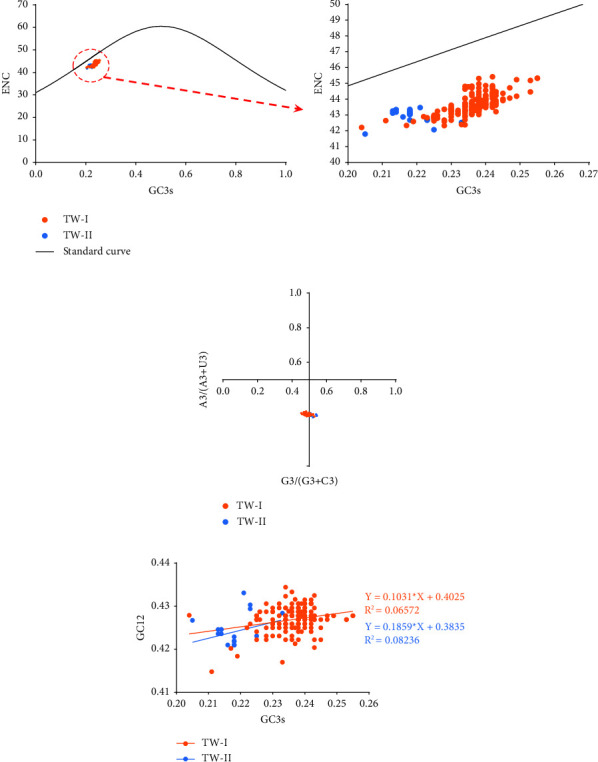
Codon usage pattern of *S*1 genes from lineage GI-7 infectious bronchitis virus (IBV) strains. (a) Effective number of codons (ENC)-plot analysis of *S*1 genes against GC3s of different sublineages. The black line represents a standard curve for when the codon usage bias is determined only by GC3s composition. (b) Parity rule 2 (PR2)-bias plot [*A*3/(*A*3+*U*3) against *G*3/(*G*3+*C*3)] of *S*1 genes. (c) Neutrality analysis (GC12 against GC3s) of *S*1 genes.

A standard curve representing the ENC values that would result if GC composition was the only factor influencing codon usage bias is shown in Figure 7(a) [44]. If the ENC value for a genome lies on the standard curve, it indicates that codon usage bias is affected only by mutation pressure [45]. Hence, the ENC result indicated that in addition to mutation pressure, other factors, such as natural selection, affected the codon usage bias of the *S*1 gene of lineage GI-7 IBVs (Figure 7(a)). In the PR2 plot, all points were separated from the center of the plot, suggesting that codon usage bias of *S*1 genes of lineage GI-7 IBVs might be determined by multiple factors, including natural selection and mutation pressure (Figure 7(b)). The neutrality analysis revealed a narrow distribution and low GC3s values (0.205–0.255). Regression analyses were performed to decipher the effects of mutational pressure and natural selection on the two sublineages of GI-7. The slopes of the TW-I and TW-II groups were 0.1031 and 0.1859, respectively, indicating that the influence of mutation pressure on codon usage bias in S1 was 10.31% and 18.59% for the TW-I and TW-II IBVs, respectively (Figure 7(c)).

## 4. Discussion

As a large family, coronaviruses are widely found in nature. A growing body of research suggests that SARS-CoV-2, which caused a global outbreak in late 2019, might have originated in nature [46]. Therefore, the study of coronaviruses in different species has received more attention from the scientific community. Chicken-derived coronavirus (IBV) is widespread worldwide, causing huge losses and threats to the poultry industry. In this study, an epidemiological surveillance of IBV conducted in China isolated 19 IBV strains from 591 infected chicken tissue samples from January 2021 to June 2022. Compared to the analyses by Lian et al. [12] in South China between April 2019 and March 2020 (139/420) and Xu et al. [11] in China from January 2016 to December 2017 (213/801), the IBV isolation rate reported in the current study decreased significantly. A credible explanation for this result is that the source of the clinical samples we collected included chickens that were not suffering from respiratory diseases due to IBV infection. We also isolated other pathogens, including avian influenza virus (AIV), Newcastle disease virus (NDV), adenovirus, and others, especially AIV-mixed infections. These other pathogens may have caused the disappearance of IBV in the process of chicken embryo passage. These potential variables within the collected samples will be addressed in a future study. Overall, our results showed that lineage GI-19 (QX type) IBV still dominated the epidemic, followed by lineage GI-7 (TW type) IBV. Other IBV lineages were sporadically isolated, which was similar to the results from research in China reported in recent years [11, 23]. However, more IBV strains were isolated in South China, with nine in Guangdong province and four in Guangxi province, which is directly linked to the development of the poultry industry in these two provinces. It is worth mentioning that our previous studies showed that lineage GVI-1 IBVs were widely prevalent in South China [14], but no GVI-1 strain was isolated from this region in this study. However, attention should be paid to this IBV lineage. The IB vaccines currently used in China are mainly attenuated vaccines of MASS strains such as H120, which are different in serotype and genotype from the popular strains such as lineages GI-19 and GI-7. Therefore, these vaccines cannot provide effective cross-protection, making it critical to conduct continuous epidemiological surveillance against IBVs and to screen for the most prevalent genotypes and serotypes to identify vaccine candidates for IB prevention and control in China.

Recombination has been an important contributing factor to the emergence and evolution of IBV, and even to the emergence of new coronaviruses and novel diseases [8, 47]. Recombination always occurs when the viral polymerase switches from one template to another during genomic synthesis in the host [48]. Therefore, the degree of recombination observed in the *S* protein, which has conformation-dependent epitopes that induce viral neutralization and serotype-specific antibodies, may be one of the main mechanisms responsible for generating genetic and antigenic diversity in IBVs [5, 49]. We identified four recombinant events of the *S*1 gene, among which lineages GI-7 and GI-13 IBVs were more frequently involved in recombination (Figure 2), indicating that these major prevalent lineages might play key roles in the transmission of IBV in China. However, the results of genome-wide and *S*1 gene recombination analyses are not always consistent [49, 50]. Hence, recombination analyses of the entire genome of IBV are equally important to deduce the epidemic evolution of the virus to a certain extent and provide a scientific basis for the prevention and control of the disease caused by IBVs.

In the last decade, lineage GI-7 IBV has become one of the most prevalent IBV lineages in China, and its influence on the poultry industry has become increasingly important [12, 23, 51]. Our results also suggest that more attention should be paid to lineage GI-7 IBV. To the best of our knowledge, this is the first reported phylodynamic analysis of all *S*1 gene sequences (*n* ≥ 1620 nt) of the lineage GI-7 IBVs uploaded to GenBank. Although some recent studies have included phylogenetic analyses of IBVs, they only focused on lineage GI-19 strains [21, 50], which do not represent the genetic evolution of other lineages of IBVs. Our Bayesian skyline plot analysis showed a sharp decline in the relative genetic density of lineage GI-7 IBVs after 2007, which could have been caused by poor regional sampling and the ban on interprovincial trade of live poultry. As avian influenza became a public health issue in China in the early 2000s [52, 53], China implemented a ban on interprovincial live poultry trade, which greatly reduced the chances of live poultry coming into contact with each other from different areas. High numbers of IBV strains were isolated after 2010, and the relative genetic density of this virus was restored, indicating that lineage GI-7 IBVs have not been effectively controlled under current prevention and control conditions. In the MCC trees, the viruses isolated from the Taiwan province were located at the root of the tree with the highest root state posterior probability of 0.98, indicating that Taiwan might have been the origin of GI-7 IBVs, which is consistent with current understanding. However, it is worth noting that some factors, such as sparse sampling, biased collection, and sequencing process, could potentially affect these conclusions [17, 54, 55]. The TW-I and TW-II sublineage TMRCAs were 1947 and 1886, respectively, which were far earlier than the first isolation of the GI-7 IBV strain (1964), indicating that there may have been unknown transmission in the intervening decades. Our results showed that after lineage GI-7 IBVs spread into South China in the 1990s, the region gradually became the transmission center of the virus, further indicating that South China played an important role in the transmission of IBVs. Moreover, our previous studies and those of others have shown that South China plays a key role in the transmission of AIV and NDV, and this region is also an important habitat for migratory birds [17, 56]. Therefore, the epidemic potential of various avian viruses in South China is complex and needs to be continuously monitored. Currently, lineage GI-7 IBVs are endemic only in China; however, with the massive development of China's poultry industry and the increase in international trade, and the risk of this virus being exported has increased. Thus, based on the One Health Model [57], a global multiregional epidemiological investigation of IBVs should be conducted to better understand IBVs.

Detailed genetic analyses of viruses are important for understanding and estimating the risk of ongoing viral transmission, as well as for developing effective countermeasures. However, few studies have focused on codon bias analysis of IBVs. Our analysis showed that the S1 genes of lineage IBVs exhibited high A/U content at the third position of synonymous codons, which was consistent with the RSCU analysis. Additionally, the ENC value was higher than 35, indicating that *S*1 genes of lineage GI-7 IBVs exhibited low codon bias. These results are consistent with a previous study that examined samples that included all IBV sequences uploaded to GenBank [44]. Other studies have also reported low codon usage bias in other viruses, including multiple types of influenza viruses and NDV, which might allow viruses to replicate in the host environment by avoiding competition and reducing the energy required for viral protein biosynthesis [29, 58–60]. Here, RSCU, ENC-plot, and PR2 analyses suggested that the bias in the *S*1 genes of lineage IBVs was influenced by mutation pressure and natural selection. Moreover, a neutrality plot analysis suggested that natural selection was the most dominant of these factors, and that sublineage TW-I IBVs were affected more by natural selection than were TW-II IBVs. This might be due to the low codon usage bias in lineage GI-7 IBVs caused by natural selection when the viruses try to adapt to host cells.

In conclusion, this study is one of the first to reveal lineage GI-7 IBV diffusion patterns among different geographic regions. Moreover, we analyzed the codon usage pattern of *S*1 genes of lineage GI-7 IBVs to provide a better understanding of the evolutionary changes in IBVs and the influence of natural selection on this virus. The findings of this study improve our understanding of IBVs and strategies for prevention and control of IBVs.

## Figures and Tables

**Table 1:** Statistically supported migration rates of lineage GI-7 IBV strains estimated from the *S*1 gene.

From	To	Mean transition rate	BF^†^	Posterior probability^‡^
Northeast	East	0.874	6.64580713	0.5574929
Taiwan	Northeast	0.831	6.77522664	0.5622454
Taiwan	South	0.957	7.42901484	0.58477348
South	Northeast	0.957	7.55680915	0.58890878
East	Central	1.130	14.1735003	0.72876805
Central	Southwest	0.803	16.6563502	0.75947414
Northeast	North	0.757	17.0458349	0.76367115
Southwest	East	1.556	20.1349931	0.79240217
Southwest	Central	0.881	31.4690196	0.85643748
South	Southwest	1.675	2843.61705	0.99814838
South	East	1.607	10678.0704	0.99950623
South	Central	1.573	85461.4888	0.99993828

^†^Bayes factor (BF) > 100 indicates decisive support for transition between hosts. Only statistically supported transitions with a BF > 3 are shown. ^‡^Posterior probability >0.5 indicates well-supported viral transition.

## Data Availability

The data that support the findings of this study are available from the corresponding author upon reasonable request.

## Supplementary Materials

Supplementary Materials Supplemental Table S1: Strain information and the composition of the codon parameters of S1 genes of lineage GI-7 IBVs. Supplemental Table S2: Strain information of IBV isolated in this study. Supplemental Table S3: Recombinant breakpoints, genes, and major and minor related sequences of genomic recombination events. Supplemental Table S4: The RSCU value of 59 codons encoding 19 amino acids according to two sublineages of S1 gene of lineage GI-7 IBV. The preferred synonymous codons are shown in bold.

## References

[1] Feikin D. R., Higdon M. M., Abu-Raddad L. J. (2022). Duration of effectiveness of vaccines against SARS-CoV-2 infection and COVID-19 disease: results of a systematic review and meta-regression. *The Lancet*.

[2] Zhu N., Zhang D., Wang W. (2020). A novel coronavirus from patients with pneumonia in China, 2019. *New England Journal of Medicine*.

[3] Sjaak de Wit J. J., Cook J. K., van der Heijden H. M. (2011). Infectious bronchitis virus variants: a review of the history, current situation and control measures. *Avian Pathology*.

[4] Cavanagh D. (2005). Coronaviruses in poultry and other birds. *Avian Pathology*.

[5] Cavanagh D. (2007). Coronavirus avian infectious bronchitis virus. *Veterinary Research*.

[6] Cavanagh D. (1983). Coronavirus IBV glycopolypeptides: size of their polypeptide moieties and nature of their oligosaccharides. *Journal of General Virology*.

[7] Shang J., Zheng Y., Yang Y. (2018). Cryo-EM structure of infectious bronchitis coronavirus spike protein reveals structural and functional evolution of coronavirus spike proteins. *PLoS Pathogens*.

[8] Ma T., Xu L., Ren M. (2019). Novel genotype of infectious bronchitis virus isolated in China. *Veterinary Microbiology*.

[9] Valastro V., Holmes E. C., Britton P. (2016). S1 gene-based phylogeny of infectious bronchitis virus: an attempt to harmonize virus classification. *Infection, Genetics and Evolution*.

[10] Feng K., Wang F., Xue Y. (2017). Epidemiology and characterization of avian infectious bronchitis virus strains circulating in southern China during the period from 2013-2015. *Scientific Reports*.

[11] Xu L., Han Z., Jiang L., Sun J., Zhao Y., Liu S. (2018). Genetic diversity of avian infectious bronchitis virus in China in recent years. *Infection, Genetics and Evolution*.

[12] Lian J., Wang Z., Xu Z. (2021). Distribution and molecular characterization of avian infectious bronchitis virus in southern China. *Poultry Science*.

[13] Zhao Y., Zhang H., Zhao J., Zhong Q., Jin J. H., Zhang G. Z. (2016). Evolution of infectious bronchitis virus in China over the past two decades. *Journal of General Virology*.

[14] Chen L., Xiang B., Hong Y. (2021). Phylogenetic analysis of infectious bronchitis virus circulating in southern China in 2016-2017 and evaluation of an attenuated strain as a vaccine candidate. *Archives of Virology*.

[15] Ren M., Sheng J., Ma T. (2019). Molecular and biological characteristics of the infectious bronchitis virus TC07-2/GVI-1 lineage isolated in China. *Infection, Genetics and Evolution*.

[16] Sun L., Tang X., Qi J. (2021). Two newly isolated GVI lineage infectious bronchitis viruses in China show unique molecular and pathogenicity characteristics. *Infection, Genetics and Evolution*.

[17] Chen L., Song J., Liu H. (2021). Phylodynamic analyses of class I Newcastle disease virus isolated in China. *Transboundary and Emerging Diseases*.

[18] Lemey P., Rambaut A., Drummond A. J., Suchard M. A. (2009). Bayesian phylogeography finds its roots. *PLoS Computational Biology*.

[19] Sun Y. K., Han X. L., Wei Y. F. (2019). Phylogeography, phylodynamics and the recent outbreak of lineage 3 porcine reproductive and respiratory syndrome viruses in China. *Transbound Emerg Dis*.

[20] Xie P., Chen L., Zhang Y. (2020). Evolutionary dynamics and age-dependent pathogenesis of sub-genotype VI.2.1.1.2.2 PPMV-1 in pigeons. *Viruses*.

[21] Fan W., Chen J., Zhang Y. (2022). Phylogenetic and spatiotemporal analyses of the complete genome sequences of avian coronavirus infectious bronchitis virus in China during 1985-2020: revealing coexistence of multiple transmission chains and the origin of LX4-type virus. *Frontiers in Microbiology*.

[22] Huang Y. P., Lee H. C., Cheng M. C., Wang C. H. (2004). S1 and N gene analysis of avian infectious bronchitis viruses in Taiwan. *Avian Diseases*.

[23] Ji J., Gao Y., Chen Q. (2020). Epidemiological investigation of avian infectious bronchitis and locally determined genotype diversity in central China: a 2016-2018 study. *Poultry Science*.

[24] Grantham R., Gautier C., Gouy M., Mercier R., Pave A. (1980). Codon catalog usage and the genome hypothesis. *Nucleic Acids Research*.

[25] Martín A., Bertranpetit J., Oliver J. L., Medina J. R. (1989). Variation in G + C-content and codon choice: differences among synonymous codon groups in vertebrate genes. *Nucleic Acids Research*.

[26] van Hemert F., van der Kuyl A. C., Berkhout B. (2016). Impact of the biased nucleotide composition of viral RNA genomes on RNA structure and codon usage. *Journal of General Virology*.

[27] Butt A. M., Nasrullah I., Qamar R., Tong Y. (2016). Evolution of codon usage in Zika virus genomes is host and vector specific. *Emerging Microbes & Infections*.

[28] Nasrullah I., Butt A. M., Tahir S., Idrees M., Tong Y. (2015). Genomic analysis of codon usage shows influence of mutation pressure, natural selection, and host features on Marburg virus evolution. *BMC Evolutionary Biology*.

[29] Yan Z., Wang R., Zhang L. (2019). Evolutionary changes of the novel Influenza D virus hemagglutinin-esterase fusion gene revealed by the codon usage pattern. *Virulence*.

[30] Yao H., Chen M., Tang Z. (2019). Analysis of synonymous codon usage bias in flaviviridae virus. *BioMed Research International*.

[31] Martin D. P. (2009). Recombination detection and analysis using RDP3. *Methods in Molecular Biology*.

[32] Katoh K., Standley D. M. (2013). MAFFT multiple sequence alignment software version 7: improvements in performance and usability. *Molecular Biology and Evolution*.

[33] Kalyaanamoorthy S., Minh B. Q., Wong T. K. F., von Haeseler A., Jermiin L. S. (2017). ModelFinder: fast model selection for accurate phylogenetic estimates. *Nature Methods*.

[34] Nguyen L. T., Schmidt H. A., von Haeseler A., Minh B. Q. (2015). IQ-TREE: a fast and effective stochastic algorithm for estimating maximum-likelihood phylogenies. *Molecular Biology and Evolution*.

[35] Rambaut A., Lam T. T., Max Carvalho L., Pybus O. G. (2016). Exploring the temporal structure of heterochronous sequences using TempEst (formerly Path-O-Gen). *Virus Evol*.

[36] Suchard M. A., Lemey P., Baele G., Ayres D. L., Drummond A. J., Rambaut A. (2018). Bayesian phylogenetic and phylodynamic data integration using BEAST 1.10. *Virus Evolution*.

[37] Rambaut A., Drummond A. J., Xie D., Baele G., Suchard M. A. (2018). Posterior summarization in bayesian phylogenetics using tracer 1.7. *Systematic Biology*.

[38] Bielejec F., Baele G., Vrancken B., Suchard M. A., Rambaut A., Lemey P. (2016). SpreaD3: interactive visualization of spatiotemporal history and trait evolutionary processes. *Molecular Biology and Evolution*.

[39] Sharp P. M., Li W. H. (1986). An evolutionary perspective on synonymous codon usage in unicellular organisms. *Journal of Molecular Evolution*.

[40] Zhou T., Gu W., Ma J., Sun X., Lu Z. (2005). Analysis of synonymous codon usage in H5N1 virus and other influenza A viruses. *Biosystems*.

[41] Wong E. H., Smith D. K., Rabadan R., Peiris M., Poon L. L. (2010). Codon usage bias and the evolution of influenza A viruses. Codon usage biases of influenza virus. *BMC Evolutionary Biology*.

[42] Sueoka N. (1995). Intrastrand parity rules of DNA base composition and usage biases of synonymous codons. *Journal of Molecular Evolution*.

[43] Sueoka N. (1988). Directional mutation pressure and neutral molecular evolution. *Proceedings of the National Academy of Sciences of the U S A*.

[44] Franzo G., Tucciarone C. M., Legnardi M., Cecchinato M. (2021). Effect of genome composition and codon bias on infectious bronchitis virus evolution and adaptation to target tissues. *BMC Genomics*.

[45] Fuglsang A. (2008). Impact of bias discrepancy and amino acid usage on estimates of the effective number of codons used in a gene, and a test for selection on codon usage. *Gene*.

[46] Temmam S., Vongphayloth K., Baquero E. (2022). Bat coronaviruses related to SARS-CoV-2 and infectious for human cells. *Nature*.

[47] Lee C. W., Jackwood M. W. (2000). Evidence of genetic diversity generated by recombination among avian coronavirus IBV. *Archives of Virology*.

[48] Worobey M., Holmes E. C. (1999). Evolutionary aspects of recombination in RNA viruses. *Journal of General Virology*.

[49] Thor S. W., Hilt D. A., Kissinger J. C., Paterson A. H., Jackwood M. W. (2011). Recombination in avian gamma-coronavirus infectious bronchitis virus. *Viruses*.

[50] Franzo G., Massi P., Tucciarone C. M. (2017). Think globally, act locally: phylodynamic reconstruction of infectious bronchitis virus (IBV) QX genotype (GI-19 lineage) reveals different population dynamics and spreading patterns when evaluated on different epidemiological scales. *PLoS One*.

[51] Feng K., Xue Y., Wang F., Chen F., Shu D., Xie Q. (2014). Analysis of S1 gene of avian infectious bronchitis virus isolated in southern China during 2011-2012. *Virus Genes*.

[52] Liu J., Xiao H., Lei F. (2005). Highly pathogenic H5N1 influenza virus infection in migratory birds. *Science*.

[53] Yu H., Gao Z., Feng Z. (2008). Clinical characteristics of 26 human cases of highly pathogenic avian influenza A (H5N1) virus infection in China. *PLoS One*.

[54] Bahl J., Pham T. T., Hill N. J. (2016). Ecosystem interactions underlie the spread of avian influenza A viruses with pandemic potential. *PLoS Pathogens*.

[55] Magee D., Suchard M. A., Scotch M. (2017). Bayesian phylogeography of influenza A/H3N2 for the 2014-15 season in the United States using three frameworks of ancestral state reconstruction. *PLoS Computational Biology*.

[56] Xiang B., Song J., Chen L. (2021). Duck-origin H5N6 avian influenza viruses induce different pathogenic and inflammatory effects in mice. *Transbound Emerg Dis*.

[57] Su S., Grdnay G. C., Lu J., Liao M., Zhang G., Li S. (2014). New “One Health” strategies needed for detection and control of emerging pathogens at Cantonese live animal markets, China. *Clinical Infectious Diseases*.

[58] Anhlan D., Grundmann N., Makalowski W., Ludwig S., Scholtissek C. (2011). Origin of the 1918 pandemic H1N1 influenza A virus as studied by codon usage patterns and phylogenetic analysis. *RNA*.

[59] Kumar N., Bera B. C., Greenbaum B. D. (2016). Revelation of influencing factors in overall codon usage bias of equine influenza viruses. *PLoS One*.

[60] Xiang B., Chen L., Cai J. (2020). Insights into genomic epidemiology, evolution, and transmission dynamics of genotype VII of class II Newcastle disease virus in China. *Pathogens*.

