# Supplementary material for: Insights into the Epidemiology, Phylodynamics, and Evolutionary Changes of Lineage GI-7 Infectious Bronchitis Virus
Source: Transbound Emerg Dis. 2023 May 16;2023:9520616. doi: 10.1155/2023/9520616 (PMC12016960; doi:10.1155/2023/9520616)
Supplement: Supplementary Materials — Supplemental Table S1: Strain information and the composition of the codon parameters of S1 genes of lineage GI-7 IBVs. Supplemental Table S2: Strain information of IBV isolated in this study. Supplemental Table S3: Recombinant breakpoints, genes, and major and minor related sequences of genomic recombination events. Supplemental Table S4: The RSCU value of 59 codons encoding 19 amino acids according to two sublineages of S1 gene of lineage GI-7 IBV. The preferred synonymous codons are shown in bold. [file 9520616.f1.docx]

**Table S1.** Strain information and the composition of the codon parameters of S1 genes of lineage GI-7 IBVs.

Sub-lineage TW-I

| SEQUENCES \ PARAMETERS | %A | %C | %U | %G | %GC | %AU | %GC1 | %GC2 | %GC12 | %GC3 | %AU3 | %A3 | %C3 | %U3 | %G3 | U3s | C3s | A3s | G3s | GC3s | ENC |
| --- | --- | --- | --- | --- | --- | --- | --- | --- | --- | --- | --- | --- | --- | --- | --- | --- | --- | --- | --- | --- | --- |
| AY606320_TW_1964 | 27.46 | 16.33 | 36.86 | 19.36 | 35.68 | 64.32 | 42.67 | 42.67 | 42.67 | 21.71 | 78.29 | 22.08 | 10.39 | 56.22 | 11.32 | 0.6352 | 0.1174 | 0.3199 | 0.1510 | 0.205 | 41.80 |
| AY606322_TW_2002 | 27.76 | 16.79 | 35.56 | 19.89 | 36.68 | 63.32 | 42.94 | 43.68 | 43.31 | 23.42 | 76.58 | 22.86 | 10.78 | 53.72 | 12.64 | 0.6097 | 0.1224 | 0.3289 | 0.1671 | 0.221 | 43.47 |
| EU822338_TW_2004 | 27.63 | 16.98 | 35.56 | 19.83 | 36.80 | 63.20 | 42.38 | 43.31 | 42.84 | 24.72 | 75.28 | 21.93 | 12.08 | 53.35 | 12.64 | 0.6004 | 0.1360 | 0.3224 | 0.1681 | 0.233 | 42.53 |
| DQ646404_TW_1995 | 28.13 | 16.54 | 35.94 | 19.39 | 35.94 | 64.06 | 41.45 | 43.12 | 42.29 | 23.23 | 76.77 | 22.68 | 11.52 | 54.09 | 11.71 | 0.6101 | 0.1300 | 0.3324 | 0.1532 | 0.218 | 43.25 |
| HM194639_LN_Northeast_2009 | 28.40 | 16.42 | 35.37 | 19.81 | 36.23 | 63.77 | 40.74 | 43.89 | 42.31 | 24.07 | 75.93 | 23.15 | 11.85 | 52.78 | 12.22 | 0.6013 | 0.1350 | 0.3369 | 0.1590 | 0.225 | 42.07 |
| HM194676_JL_Northeast_2009 | 28.00 | 16.42 | 36.12 | 19.45 | 35.87 | 64.13 | 41.26 | 43.12 | 42.19 | 23.23 | 76.77 | 22.68 | 11.52 | 54.09 | 11.71 | 0.6101 | 0.1300 | 0.3333 | 0.1536 | 0.218 | 43.35 |
| JF330898_HB_North_2010 | 28.00 | 16.48 | 36.12 | 19.39 | 35.87 | 64.13 | 41.26 | 43.12 | 42.19 | 23.23 | 76.77 | 22.68 | 11.52 | 54.09 | 11.71 | 0.6101 | 0.1300 | 0.3352 | 0.1545 | 0.218 | 43.12 |
| JQ739246_HB_North_2011 | 28.00 | 16.48 | 36.12 | 19.39 | 35.87 | 64.13 | 41.26 | 43.12 | 42.19 | 23.23 | 76.77 | 22.68 | 11.52 | 54.09 | 11.71 | 0.6101 | 0.1300 | 0.3352 | 0.1545 | 0.218 | 43.12 |
| JQ739299_HLJ_Northeast_2011 | 27.94 | 16.36 | 36.31 | 19.39 | 35.75 | 64.25 | 41.08 | 43.12 | 42.10 | 23.05 | 76.95 | 22.49 | 11.34 | 54.46 | 11.71 | 0.6143 | 0.1279 | 0.3324 | 0.1545 | 0.216 | 42.88 |
| JQ739366_SD_East_2011 | 28.00 | 16.70 | 36.13 | 19.18 | 35.88 | 64.12 | 41.71 | 43.20 | 42.46 | 22.72 | 77.28 | 22.72 | 11.36 | 54.56 | 11.36 | 0.6168 | 0.1284 | 0.3306 | 0.1474 | 0.213 | 43.25 |
| JQ739374_SD_East_2011 | 28.13 | 16.48 | 36.06 | 19.33 | 35.81 | 64.19 | 41.26 | 42.94 | 42.10 | 23.23 | 76.77 | 22.68 | 11.52 | 54.09 | 11.71 | 0.6101 | 0.1300 | 0.3352 | 0.1550 | 0.218 | 43.03 |
| JQ764816_GX_South_1988 | 27.63 | 16.48 | 35.87 | 20.01 | 36.49 | 63.51 | 43.12 | 42.75 | 42.94 | 23.61 | 76.39 | 22.30 | 10.78 | 54.09 | 12.83 | 0.6152 | 0.1226 | 0.3252 | 0.1729 | 0.223 | 42.70 |
| JQ764828_GX_South_1988 | 27.57 | 16.54 | 35.87 | 20.01 | 36.56 | 63.44 | 43.12 | 42.94 | 43.03 | 23.61 | 76.39 | 22.30 | 10.78 | 54.09 | 12.83 | 0.6152 | 0.1226 | 0.3243 | 0.1724 | 0.223 | 42.67 |
| KJ524626_SC_Southwest_2012 | 28.12 | 16.64 | 36.06 | 19.18 | 35.82 | 64.18 | 41.53 | 43.20 | 42.36 | 22.72 | 77.28 | 22.72 | 11.36 | 54.56 | 11.36 | 0.6168 | 0.1284 | 0.3280 | 0.1503 | 0.214 | 43.18 |
| KU364609_SC_Southwest_2013 | 28.07 | 16.42 | 36.12 | 19.39 | 35.81 | 64.19 | 41.45 | 42.75 | 42.10 | 23.23 | 76.77 | 22.86 | 11.52 | 53.90 | 11.71 | 0.6080 | 0.1300 | 0.3361 | 0.1541 | 0.218 | 42.68 |
| MG448607_HB_North_2012 | 28.00 | 16.48 | 36.12 | 19.39 | 35.87 | 64.13 | 41.08 | 43.31 | 42.19 | 23.23 | 76.77 | 22.68 | 11.52 | 54.09 | 11.71 | 0.6101 | 0.1300 | 0.3352 | 0.1545 | 0.218 | 43.23 |
| MH397172_FJ_East_2017 | 28.06 | 16.64 | 36.13 | 19.18 | 35.82 | 64.18 | 41.53 | 43.20 | 42.36 | 22.72 | 77.28 | 22.72 | 11.36 | 54.56 | 11.36 | 0.6181 | 0.1287 | 0.3297 | 0.1474 | 0.213 | 43.12 |
| MN509343_HuB_Central_2018 | 28.06 | 16.70 | 36.00 | 19.24 | 35.94 | 64.06 | 41.71 | 43.20 | 42.46 | 22.91 | 77.09 | 22.72 | 11.36 | 54.38 | 11.55 | 0.6160 | 0.1287 | 0.3288 | 0.1499 | 0.214 | 43.34 |
| MN509380_HuB_Central_2018 | 28.06 | 16.70 | 36.00 | 19.24 | 35.94 | 64.06 | 41.71 | 43.20 | 42.46 | 22.91 | 77.09 | 22.72 | 11.36 | 54.38 | 11.55 | 0.6160 | 0.1287 | 0.3288 | 0.1499 | 0.214 | 43.34 |
| MN531555_SD_East_2011 | 28.00 | 16.48 | 36.18 | 19.33 | 35.81 | 64.19 | 41.08 | 43.12 | 42.10 | 23.23 | 76.77 | 22.68 | 11.52 | 54.09 | 11.71 | 0.6101 | 0.1300 | 0.3352 | 0.1545 | 0.218 | 43.10 |
| MN531556_SD_East_2011 | 28.13 | 16.48 | 36.06 | 19.33 | 35.81 | 64.19 | 41.26 | 42.94 | 42.10 | 23.23 | 76.77 | 22.68 | 11.52 | 54.09 | 11.71 | 0.6101 | 0.1300 | 0.3352 | 0.1550 | 0.218 | 43.03 |
| MN531554_LN_Northeast_2012 | 27.94 | 16.36 | 36.25 | 19.45 | 35.81 | 64.19 | 41.26 | 42.94 | 42.10 | 23.23 | 76.77 | 22.68 | 11.52 | 54.09 | 11.71 | 0.6101 | 0.1300 | 0.3361 | 0.1550 | 0.218 | 43.13 |

Sub-lineage TW-II

| SEQUENCES \ PARAMETERS | %A | %C | %U | %G | %GC | %AU | %GC1 | %GC2 | %GC12 | %GC3 | %AU3 | %A3 | %C3 | %U3 | %G3 | U3s | C3s | A3s | G3s | GC3s | ENC |
| --- | --- | --- | --- | --- | --- | --- | --- | --- | --- | --- | --- | --- | --- | --- | --- | --- | --- | --- | --- | --- | --- |
| AY606320_TW_1964 | 27.46 | 16.33 | 36.86 | 19.36 | 35.68 | 64.32 | 42.67 | 42.67 | 42.67 | 21.71 | 78.29 | 22.08 | 10.39 | 56.22 | 11.32 | 0.6352 | 0.1174 | 0.3199 | 0.1510 | 0.205 | 41.80 |
| AY606322_TW_2002 | 27.76 | 16.79 | 35.56 | 19.89 | 36.68 | 63.32 | 42.94 | 43.68 | 43.31 | 23.42 | 76.58 | 22.86 | 10.78 | 53.72 | 12.64 | 0.6097 | 0.1224 | 0.3289 | 0.1671 | 0.221 | 43.47 |
| EU822338_TW_2004 | 27.63 | 16.98 | 35.56 | 19.83 | 36.80 | 63.20 | 42.38 | 43.31 | 42.84 | 24.72 | 75.28 | 21.93 | 12.08 | 53.35 | 12.64 | 0.6004 | 0.1360 | 0.3224 | 0.1681 | 0.233 | 42.53 |
| DQ646404_TW_1995 | 28.13 | 16.54 | 35.94 | 19.39 | 35.94 | 64.06 | 41.45 | 43.12 | 42.29 | 23.23 | 76.77 | 22.68 | 11.52 | 54.09 | 11.71 | 0.6101 | 0.1300 | 0.3324 | 0.1532 | 0.218 | 43.25 |
| HM194639_LN_Northeast_2009 | 28.40 | 16.42 | 35.37 | 19.81 | 36.23 | 63.77 | 40.74 | 43.89 | 42.31 | 24.07 | 75.93 | 23.15 | 11.85 | 52.78 | 12.22 | 0.6013 | 0.1350 | 0.3369 | 0.1590 | 0.225 | 42.07 |
| HM194676_JL_Northeast_2009 | 28.00 | 16.42 | 36.12 | 19.45 | 35.87 | 64.13 | 41.26 | 43.12 | 42.19 | 23.23 | 76.77 | 22.68 | 11.52 | 54.09 | 11.71 | 0.6101 | 0.1300 | 0.3333 | 0.1536 | 0.218 | 43.35 |
| JF330898_HB_North_2010 | 28.00 | 16.48 | 36.12 | 19.39 | 35.87 | 64.13 | 41.26 | 43.12 | 42.19 | 23.23 | 76.77 | 22.68 | 11.52 | 54.09 | 11.71 | 0.6101 | 0.1300 | 0.3352 | 0.1545 | 0.218 | 43.12 |
| JQ739246_HB_North_2011 | 28.00 | 16.48 | 36.12 | 19.39 | 35.87 | 64.13 | 41.26 | 43.12 | 42.19 | 23.23 | 76.77 | 22.68 | 11.52 | 54.09 | 11.71 | 0.6101 | 0.1300 | 0.3352 | 0.1545 | 0.218 | 43.12 |
| JQ739299_HLJ_Northeast_2011 | 27.94 | 16.36 | 36.31 | 19.39 | 35.75 | 64.25 | 41.08 | 43.12 | 42.10 | 23.05 | 76.95 | 22.49 | 11.34 | 54.46 | 11.71 | 0.6143 | 0.1279 | 0.3324 | 0.1545 | 0.216 | 42.88 |
| JQ739366_SD_East_2011 | 28.00 | 16.70 | 36.13 | 19.18 | 35.88 | 64.12 | 41.71 | 43.20 | 42.46 | 22.72 | 77.28 | 22.72 | 11.36 | 54.56 | 11.36 | 0.6168 | 0.1284 | 0.3306 | 0.1474 | 0.213 | 43.25 |
| JQ739374_SD_East_2011 | 28.13 | 16.48 | 36.06 | 19.33 | 35.81 | 64.19 | 41.26 | 42.94 | 42.10 | 23.23 | 76.77 | 22.68 | 11.52 | 54.09 | 11.71 | 0.6101 | 0.1300 | 0.3352 | 0.1550 | 0.218 | 43.03 |
| JQ764816_GX_South_1988 | 27.63 | 16.48 | 35.87 | 20.01 | 36.49 | 63.51 | 43.12 | 42.75 | 42.94 | 23.61 | 76.39 | 22.30 | 10.78 | 54.09 | 12.83 | 0.6152 | 0.1226 | 0.3252 | 0.1729 | 0.223 | 42.70 |
| JQ764828_GX_South_1988 | 27.57 | 16.54 | 35.87 | 20.01 | 36.56 | 63.44 | 43.12 | 42.94 | 43.03 | 23.61 | 76.39 | 22.30 | 10.78 | 54.09 | 12.83 | 0.6152 | 0.1226 | 0.3243 | 0.1724 | 0.223 | 42.67 |
| KJ524626_SC_Southwest_2012 | 28.12 | 16.64 | 36.06 | 19.18 | 35.82 | 64.18 | 41.53 | 43.20 | 42.36 | 22.72 | 77.28 | 22.72 | 11.36 | 54.56 | 11.36 | 0.6168 | 0.1284 | 0.3280 | 0.1503 | 0.214 | 43.18 |
| KU364609_SC_Southwest_2013 | 28.07 | 16.42 | 36.12 | 19.39 | 35.81 | 64.19 | 41.45 | 42.75 | 42.10 | 23.23 | 76.77 | 22.86 | 11.52 | 53.90 | 11.71 | 0.6080 | 0.1300 | 0.3361 | 0.1541 | 0.218 | 42.68 |
| MG448607_HB_North_2012 | 28.00 | 16.48 | 36.12 | 19.39 | 35.87 | 64.13 | 41.08 | 43.31 | 42.19 | 23.23 | 76.77 | 22.68 | 11.52 | 54.09 | 11.71 | 0.6101 | 0.1300 | 0.3352 | 0.1545 | 0.218 | 43.23 |
| MH397172_FJ_East_2017 | 28.06 | 16.64 | 36.13 | 19.18 | 35.82 | 64.18 | 41.53 | 43.20 | 42.36 | 22.72 | 77.28 | 22.72 | 11.36 | 54.56 | 11.36 | 0.6181 | 0.1287 | 0.3297 | 0.1474 | 0.213 | 43.12 |
| MN509343_HuB_Central_2018 | 28.06 | 16.70 | 36.00 | 19.24 | 35.94 | 64.06 | 41.71 | 43.20 | 42.46 | 22.91 | 77.09 | 22.72 | 11.36 | 54.38 | 11.55 | 0.6160 | 0.1287 | 0.3288 | 0.1499 | 0.214 | 43.34 |
| MN509380_HuB_Central_2018 | 28.06 | 16.70 | 36.00 | 19.24 | 35.94 | 64.06 | 41.71 | 43.20 | 42.46 | 22.91 | 77.09 | 22.72 | 11.36 | 54.38 | 11.55 | 0.6160 | 0.1287 | 0.3288 | 0.1499 | 0.214 | 43.34 |
| MN531555_SD_East_2011 | 28.00 | 16.48 | 36.18 | 19.33 | 35.81 | 64.19 | 41.08 | 43.12 | 42.10 | 23.23 | 76.77 | 22.68 | 11.52 | 54.09 | 11.71 | 0.6101 | 0.1300 | 0.3352 | 0.1545 | 0.218 | 43.10 |
| MN531556_SD_East_2011 | 28.13 | 16.48 | 36.06 | 19.33 | 35.81 | 64.19 | 41.26 | 42.94 | 42.10 | 23.23 | 76.77 | 22.68 | 11.52 | 54.09 | 11.71 | 0.6101 | 0.1300 | 0.3352 | 0.1550 | 0.218 | 43.03 |
| MN531554_LN_Northeast_2012 | 27.94 | 16.36 | 36.25 | 19.45 | 35.81 | 64.19 | 41.26 | 42.94 | 42.10 | 23.23 | 76.77 | 22.68 | 11.52 | 54.09 | 11.71 | 0.6101 | 0.1300 | 0.3361 | 0.1550 | 0.218 | 43.13 |

**Table S2.** Strain information of IBV isolated in this study

| No. | Accession No. | Name | Date | Location | Host |
| --- | --- | --- | --- | --- | --- |
| 1 | OP820081 | 21B1438GXQZ | 2021 | Guangxi | Chicken |
| 2 | OP820082 | 21B1442YNKM | 2021 | Yunnan | Chicken |
| 3 | OP820083 | 21B1396GDMZ | 2021 | Guangdong | Chicken |
| 4 | OP820084 | 21B620GDYF | 2021 | Guangdong | Chicken |
| 5 | OP820085 | 21B1388GXQZ | 2021 | Guangxi | Chicken |
| 6 | OP820086 | 21B01GDHZ | 2021 | Guangdong | Chicken |
| 7 | OP820087 | 21B666HBHD | 2021 | Hebei | Chicken |
| 8 | OP820088 | 21B1139GDJM | 2021 | Guangdong | Chicken |
| 9 | OP820089 | 21B1042YNKM | 2021 | Yunnan | Chicken |
| 10 | OP820090 | 21B1336GDJM | 2021 | Guangdong | Chicken |
| 11 | OP820091 | 21B1200GDJM | 2021 | Guangdong | Chicken |
| 12 | OP820092 | 21B590JXGZ | 2021 | Jiangxi | Chicken |
| 13 | OP820093 | 21B1655YNKM | 2021 | Yunnan | Chicken |
| 14 | OP820094 | 22B82GXQZ | 2022 | Guangxi | Chicken |
| 15 | OP820095 | 22B45GDGZ | 2022 | Guangdong | Chicken |
| 16 | OP820096 | 22B74GDGZ | 2022 | Guangdong | Chicken |
| 17 | OP820097 | 22B04SDDZ | 2022 | Shandong | Chicken |
| 18 | OP820098 | 21B1624GXQZ | 2021 | Guangxi | Chicken |
| 19 | OP820099 | 22B188GDKP | 2022 | Guangdong | Chicken |

**Table S3.** Recombinant breakpoints, genes, and major and minor related sequences of genomic recombination events.

| Breakpoints | | Strains | Major sequence | Minor sequence | Detection method (P-value) |
| --- | --- | --- | --- | --- | --- |
| Start | End |  |  |  |  |
| 519 | 1324 | 21B1336GDJM | KX107652_CK/CH/FJ/ZZ1304_China_2013 | JQ739375_ck/CH/LSD/110857_China_2011 | RDP (1.58×10^-08) |
|  |  |  | KX107672_CK/CH/GD/LZ15_China_2015 |  | GENECONV (7.99×10^-07) |
|  |  |  |  |  | Bootscan (6.16×10^-26) |
|  |  |  |  |  | Maxchi (1.18×10^-08) |
|  |  |  |  |  | SiSscan (1.08×10^-28) |
|  |  |  |  |  | 3Seq  (1.27×10^-26) |
| 501 | 1525 | 21B1388GXQZ | AF193423_QXIBV_China_1999 | DQ167147_CK/CH/LSC/99I_China_1999 | RDP (1.38×10^-19) |
|  |  |  |  | HQ850618_GX-YL9_China_2007 | GENECONV (1.56×10^-16) |
|  |  |  |  |  | Bootscan (5.25×10^-29) |
|  |  |  |  |  | Maxchi (1.30×10^-18) |
|  |  |  |  |  | Chimaera (2.31×10^-17) |
|  |  |  |  |  | SiSscan (5.52×10^-30) |
|  |  |  |  |  | 3Seq  (1.46×10^-47) |
| 1 | 1385 | 21B590JXGZ | Z83975_UK/7/91_UnitedKingdom_1991 | DQ646405_TW2575/98_China_1998 | RDP (1.12×10^-09) |
|  |  |  |  | KX107672_CK/CH/GD/LZ15_China_2015 | GENECONV (1.19×10^-04) |
|  |  |  |  |  | Bootscan (1.02×10^-39) |
|  |  |  |  |  | Maxchi (5.55×10^-09) |
|  |  |  |  |  | SiSscan (5.07×10^-47) |
|  |  |  |  |  | 3Seq  (7.05×10^-41) |
| 1188 | 1544 | 21B1200GDJM | M99482_ARK99_USA_1973 | l14070_JMK_USA_1964 | Bootscan (3.76×10^-35) |
|  |  |  |  | L14069_Gray_USA_1960 | Maxchi (1.94×10^-06) |
|  |  |  |  |  | Chimaera (1.34×10^-06) |
|  |  |  |  |  | SiSscan (6.72×10^-42) |
|  |  |  |  |  | 3Seq  (1.24×10^-21) |

**Table S4**. The RSCU value of 59 codons encoding 19 amino acids according to two sub-lineages of S1 gene of lineage GI-7 IBV. The preferred synonymous codons are shown in bold.

| **Amino acid** | **Codon** | **Relative synonymous codon usage** | | **Amino acid** | **Codon** | **Relative synonymous codon usage** | |
| --- | --- | --- | --- | --- | --- | --- | --- |
|  |  | **TW-I** | **TW-II** |  |  | **TW-I** | **TW-II** |
| Phe | UUU(F) | **1.35** | **1.61** | Ala | GCU(A) | 1.16 | 1.52 |
|  | UUC(F) | 0.65 | 0.39 |  | GCC(A) | 0.8 | 0.63 |
| Leu | UUA(L) | **2.61** | **2.43** |  | GCA(A) | **1.85** | **1.66** |
|  | UUG(L) | 1.32 | 1.26 |  | GCG(A) | 0.18 | 0.19 |
|  | CUU(L) | 0.86 | 0.56 | Tyr | UAU(Y) | 1 | **1.32** |
|  | CUC(L) | 0.46 | 0.76 |  | UAC(Y) | 1 | 0.68 |
|  | CUA(L) | 0.63 | 0.66 | His | CAU(H) | **1.26** | **1.22** |
|  | CUG(L) | 0.12 | 0.33 |  | CAC(H) | 0.74 | 0.78 |
| Ile | AUU(I) | **1.69** | **1.74** | Gln | CAA(Q) | **1.44** | **1.5** |
|  | AUC(I) | 0.4 | 0.48 |  | CAG(Q) | 0.56 | 0.5 |
|  | AUA(I) | 0.91 | 0.78 | Asn | AAU(N) | **1.18** | **1.27** |
| Val | GUU(V) | **2.25** | **2.09** |  | AAC(N) | 0.82 | 0.73 |
|  | GUC(V) | 0.39 | 0.58 | Lys | AAA(K) | **1.1** | **1.44** |
|  | GUA(V) | 0.77 | 0.7 |  | AAG(K) | 0.9 | 0.56 |
|  | GUG(V) | 0.59 | 0.63 | Asp | GAU(D) | **1.24** | 0.76 |
| Ser | UCU(S) | **1.69** | **1.83** |  | GAC(D) | 0.76 | **1.24** |
|  | UCC(S) | 0.12 | 0.05 | Glu | GAA(E) | 0.72 | 0.51 |
|  | UCA(S) | 1.66 | 1.37 |  | GAG(E) | **1.28** | **1.49** |
|  | UCG(S) | 0.45 | 0.58 | Cys | UGU(C) | **1.23** | **1.47** |
|  | AGU(S) | 1.17 | 1.3 |  | UGC(C) | 0.77 | 0.53 |
|  | AGC(S) | 0.91 | 0.87 | Trp | UGG(W) | 1 | 1 |
| Pro | CCU(P) | **1.82** | **1.55** | Arg | CGU(R) | 0.7 | 0.7 |
|  | CCC(P) | 0.67 | 0.76 |  | CGC(R) | 0.27 | 0.04 |
|  | CCA(P) | 1.09 | 1.22 |  | CGA(R) | 0.07 | 0.26 |
|  | CCG(P) | 0.42 | 0.48 |  | CGG(R) | 0.72 | 0.28 |
| Thr | ACU(T) | **1.46** | **1.59** |  | AGA(R) | 2.03 | **2.41** |
|  | ACC(T) | 0.54 | 0.55 |  | AGG(R) | **2.22** | 2.3 |
|  | ACA(T) | **1.46** | 1.54 | Gly | GGU(G) | **2.93** | **2.64** |
|  | ACG(T) | 0.53 | 0.32 |  | GGC(G) | 0.83 | 1.1 |
|  |  |  |  |  | GGA(G) | 0.03 | 0.16 |
|  |  |  |  |  | GGG(G) | 0.2 | 0.1 |
